# Sexual and reproductive health and access: Results of a rapid epidemiological assessment among migrant peoples in transit through Darién, Panamá

**DOI:** 10.3389/frph.2022.953979

**Published:** 2022-11-29

**Authors:** Jennifer Toller Erausquin, Joanne Sánchez, Anyi Yu Pon, Mónica Jhangimal, Eugenia Millender, Yudith Peña, Winroy Ng, Adelys Reina, Candy Nakad, Joselid Quintana, Roberto Herrera Veces, Grace Vistica, Justo Pinzón-Espinosa, Gonzalo Cabezas-Talavero, Jennifer Katz, Juan Miguel Pascale, Fátima Rodríguez-Álvarez, Amanda Gabster

**Affiliations:** ^1^ Department of Public Health Education, School of Health and Human Sciences, University of North Carolina Greensboro, Greensboro, NC, United States; ^2^ Center of Population Sciences for Health Equity, College of Nursing, Florida State University, Tallahassee, FL, United States; ^3^ Complejo Hospitalario Arnufo Arias de Madrid, Panamá City, Panamá; ^4^ Gorgas Memorial Institute of Health Studies, Panamá City, Panamá; ^5^ Community Development Network of the Americas, Panamá City, Panamá; ^6^ Instituto de Medicina Tropical, Laboratorio de Protozoarios de Biología Molecular, Universidad Central de Venezuela, Caracas, Venezuela; ^7^ Tulane University School of Public Health and Tropical Medicine, New Orleans, LA, United States; ^8^ Sant Pau Mental Health Group, Institut d’Investigació Biomèdica Sant Pau (IBB-San Pau), Hospital de la San Creu i Sant Pau, Universisat Autònoma de Barcelona, Barcelona, Spain; ^9^ Department of Medicine, School of Medicine, University of Barcelona, Barcelona, Spain; ^10^ Department of Clinical Psychiatry, School of Medicine, University of Panamá, Panamá City, Panamá; ^11^ Institut d’Investigació i Innovació Parc Tauli (I3PT), Sabadell, Spain; ^12^ Faculty of Medicine, University of Panamá, Panamá City, Panamá; ^13^ National Research System of Panamá (SNI), Panamá, Panamá

**Keywords:** sexual and reproductive health (SRH), migrant health care, sexual behavior, sexually transmitted infection (STI), Latin America & Caribbean

## Abstract

**Background:**

The foot transit of migrant peoples originating from the Caribbean, South America, Asia, and Sub-Saharan Africa through the Darién Forest (DF) in Eastern Panamá towards North America has increased in recent years from approximately 30,000 people/year to >133,000 in 2021. In the DF, there is no food/housing provision nor healthcare access. Very little is known of sexual and reproductive health (SRH) among this population. This study used rapid epidemiological methods to describe the SRH situation among migrant peoples in transit through the DF.

**Methods:**

This cross-sectional study randomly selected migrant people in transit (men and women) at a Migrant Reception Station in Darién, Panamá, between January 4–11, 2022. Data collection included a self-applied questionnaire (≥18 years); clinical screening (≥12 years); and HCG, treponemal antibodies, and HIV(I/II) lateral-flow tests with blood samples (≥12 years). Descriptive analyses were used to report findings.

**Results:**

In all, 69 men and 55 women participated in the self-applied questionnaire, 70 men and 51 women in clinical screening; 78 men and 63 women in HCG, treponemal antibody and HIV testing. Overall, 26.1% (18/69) men and 36.4% (20/55) women reported sexual intercourse within the past month. The last sex partner was casual among 43.0% (21/49) of men and 27.8% (10/36) of women; of those, 42.9% (9/21) of men and 80.0% (8/10) of women reported this sex was condomless. Among women, 20.0% (11/55) tested positive for pregnancy; 5 of these pregnancies were planned. Of those screened, a reproductive tract infection symptom was reported by 5.7% (4/70) of men and 58.8% (30/51) of women. A total of 32.7% (18/55) of men and 18.2% (8/44) of women reported no prior HIV testing. Of 78 men, HIV and treponemal antibodies were found among 1.3% (*n* = 1) and 2.6% (*n* = 2), and among 63 women, 3.2% (*n* = 2) and 3.2% (*n* = 2), respectively.

**Conclusions:**

This rapid epidemiological assessment found high recent sexual activity, low condom use with casual partners, and a need for increased HIV and syphilis testing and treatment. There is a need for increased testing, condom provision, and SRH healthcare access at migrant reception stations that receive migrant peoples in transit through Panamá.

## Introduction

Global migration has greatly increased in the past four decades (1), with a particularly dramatic increase in the number of forcibly displaced people in the past 10 years (2). The current worldwide tally of migrant peoples is over 281 million, and 1 in 95 people worldwide is forcibly displaced, meaning that they had to move away from their home due to conflict, violence, or human rights violations (1). Within forcibly displaced people, the United Nations High Commissioner for Refugees specifically defines four groups as populations of concern: refugees, people in refugee-like situations, asylum-seekers, and Venezuelans displaced abroad.

International migration can be physically difficult, dangerous, and chaotic. During transit, the health needs of migrant peoples, and particularly their sexual and reproductive health needs, grow as they are faced with conditions and vulnerabilities for which they are unprepared (3). The migration process often disrupts supportive and familiar social structures, and may involve precipitous loss of financial means of support and security provided by family and friends, and disintegration of law and order (4). Migration and other humanitarian contexts create circumstances that inhibit migrant peoples' access to sexual and reproductive health (SRH) services and simultaneously increase vulnerability to sexual violence, coercion, and exploitation; transactional or survival sex; sexually transmitted infections; and unintended pregnancies (4, 5). These vulnerabilities are intensified among unauthorized or undocumented migrants and asylum seekers (6). Additionally, humanitarian settings often have very limited access to sexual and reproductive (SRH) healthcare (7).

A rapid escalation in population movement is apparent in the Americas, where migrant peoples from around the world travel through South and Central America headed to North America. Though other portions of the journey may involve traveling *via* airplane, boat, bus, or train, there is a 106-km gap in the Pan-American Highway near the Colombia-Panamá border. At this point, migrant peoples must cross on foot through the Darién Forest (DF; sometimes called the Darién Gap), a roadless, dense neotropical forest. The journey is dangerous and physically demanding; based on the route taken, food and other supplies carried, and the age and abilities of those traveling, the trek often takes 3–14 days to emerge from the DF in Panamá (8). The International Organization for Migration (UN Migration) and Panamá's National Migration Service estimated that over 133,000 migrant peoples passed through the DF in 2021, more than four times the previous highest annual count [30,000 for the entire year 2016] (9–11). In the first nine months of 2022, more than 150,000 migrant peoples passed through the DF (12).

In Panamá, migrant peoples who emerge from the DF are met by the National Border Police (SENAFRONT) and guided to Migrant Reception Stations (MRS). The MRS are part of a coordinated U.S.-Panamá effort to control the flow of migrant peoples. With the aid of U.S. tools and training, biometric measurements are taken of all migrants at the MRS to identify suspected terrorists and maintain documentation of migrating people (13). As described in our study protocol (14), time spent at the MRS varies based on the individual's nationality, demographic characteristics, and needs. Stays at the MRS can be as short as a few days for citizens of countries with expedited processes (including Cuba and Haiti), to several weeks or months.

To date, much of what is known about the healthcare needs of migrant peoples passing through the Darién Forest and the continued journey north comes from journalistic reports and anecdotes (15, 16). However, there is a dearth of information about the current state of sexual and reproductive health among migrant peoples in transit in this area. Therefore, the current study aims to describe the sexual and reproductive health of migrant peoples in transit and to identify targets for future intervention.

## Methods

### Study setting

This study was undertaken in a Migrant Reception Station (MRS) in the Darién province of eastern Panamá. The study protocol received ethical approval from the Comité de Bioética de la Investigación del Instituto Conmemorativo Gorgas de Estudios de la Salud (260/CBI/ICGES/21).

### Study participants

Participants were randomly selected at the MRS in Darién. Travel groups (typically 2–8 persons) were introduced to the study; the study objectives and methods were briefly explained. One person from adults in the travel group (≥18 years) and one person from children/youth in the travel group (<18 years) was selected randomly to be invited to participate. Participation was voluntary. Sample size was calculated by $n=\frac{Z^{2}P\left(1-P\right)}{d^{2}}$, where *n* is the sample size, *Z* the confidence statistic, *P* is expected prevalence and *d* is the precision, resulting in an intended sample of 120 participants <18 years and 160 participants ≥18 years. We expected the prevalence of pregnancy to be 10% (17); this prevalence was used for sample size calculation.

### Procedures

We obtained approval from the Ministry of Health, and the Ministry of Security (National Migration Service and the National Border Service) to work in the MRS. Participants were selected as described above. Adults of legal age (≥18 years) were taken to a place where the study objectives and procedures could be explained in private so others would not influence participation. Adults were asked to sign their own consent forms. Children (<12 years) were accompanied by an adult guardian when the study was explained; the adult guardian signed a consent form. Adolescents (12–17 years) and their guardians were explained the study in a private location, separately, and were each asked to sign an informed assent or consent form, respectively. The current analysis focused only on data from adolescents and adults. A total of 12 people withdrew from the study, including 4 minors <17 years old; their data were not analyzed here.

After receiving informed consent and assent, participants (≥18 years) self-applied a questionnaire on a tablet computer (14); participants who needed assistance had a trained study volunteer read the questions and record the participant's responses on the tablet computer. Participants then gave blood (2 EDTA tubes) and mid-flow urine (20–50 ml) samples (14), and took part in the sexual and reproductive clinical survey and exam. Additional details about data collection including the self-administered questionnaire are available in our published study protocol and supplementary materials (14).

### Laboratory methods

Blood samples were centrifuged. Lateral flow immunochromatography tests (CTK Biotech, Beijing, China) were performed for HIV (I/II), syphilis (treponemal seroreactivity) and pregnancy (HCG). HIV positive samples were tested again with a second brand (Abbott Diagnostics, Scarborough, United States), as per national guidelines. Samples with two positive results were analyzed for CD4 levels using *Vistect Advanced* (Omega Diagnostics Scotland, UK), which identifies samples with CD4 below 200 cells/mm^3^ and thereby meeting the WHO definition of advanced HIV disease (18). Mid-flow urine samples were tested for leukocyte esterase. All female participants were offered a pelvic exam; those who consented and had discharge were offered diagnosis for bacterial vaginosis (Amsel criteria) and yeast (microscopy KOH test).

### Result and treatment

Participants were offered results of testing and adequate treatment [according to national guidelines (19)] within 3 h of being included in the study. Our guidelines for on-site treatment of conditions identified as part of the study are described in our protocol paper (14). All participants were offered a meeting with a psychologist and psychiatrist.

### Statistical analyses

We conducted descriptive analyses including frequencies and percentages for categorical variables and median and interquartile range for continuous variables. For categorical variables, we also conducted bivariable analyses (Chi square tests comparing men to women) to examine potential gender differences. All analyses were conducted using Stata 17.0 (StataCorp LP, College Station, United States).

## Results

Data collection was completed at a Migrant Reception Station in Darién, Panamá, between January 4–11, 2022. During that period, the MRS housed or received 688 migrant peoples 18 years and older and 708 migrant peoples 12 years and older.

### Demographic characteristics

Results of descriptive analyses for demographic characteristics are shown in Table 1. In all, 69 men and 55 women were included in the self-applied questionnaire, 70 men and 51 women in the clinical screening and 78 men and 63 women consented/assented to laboratory testing of blood. No participants identified as transgender, nonbinary, or another gender. Participants' region of origin showed statistically significant differences by gender: Caribbean origin for 13.2% (9/68) of men and 52.8% (28/53) of women; North or South America for 51.5% (35/68) of men and 34.0% (18/53) of women; Asia for 5.6% (4/68) of men and 0.0% (0/53) of women; and Sub-Saharan Africa for 29.4% (20/68) of men and 13.2% (7/53) of women.

**Table 1:** Demographic characteristics (self-reported by adults age ≥18 years).

	Men, *n* (%)	Women, *n* (%)	*p*-value (difference between genders)
Age	*n* = 69	*n* = 55	0.86
	Median: 31 (IQR: 27–39)	Median: 30 (IQR: 27–35)	
median: 31 years (IQR: 19–56)			
18–25	14 (20.3)	12 (21.8)	
26–31	20 (29.0)	19 (34.6)	
32–39	19 (27.5)	12 (21.8)	
≥40	16 (23.2)	12 (21.8)	
Birth country	*n* = 68	*n* = 53	<0.01*
Caribbean	9 (13.2)	28 (52.8)	
South America	35 (51.5)	18 (34.0)	
Asia	4 (5.6)	0 (0.0)	
East or Central Africa	20 (29.4)	7 (13.2)	
Highest level of education	*n* = 68	*n* = 53	<0.01*
Never had formal education	9 (13.2)	28 (52.8)	
Grades 1–6 (Primary)	35 (51.5)	18 (34.0)	
Grades 7–12 (Secondary)	4 (5.9)	0 (0.0)	
Post-secondary school	20 (29.4)	7 (13.2)	
Marital status	*n* = 63	*n* = 50	<0.01*
Single, divorced, widowed, or separated	39 (61.9)	15 (30.0)	
Married or in union	24 (38.1)	35 (70.0)	
Did participant have a safe place to sleep at night in the past month?	*n* = 66	*n* = 54	0.37
Never	24 (36.4)	23 (42.6)	
Sometimes	29 (43.9)	17 (31.5)	
Always	13 (19.7)	14 (25.9)	
Main reason for travelling	*n* = 66	*n* = 55	0.11
War or conflict	22 (33.3)	9 (16.4)	
Other violence	11 (16.7)	11 (20.0)	
Work	8 (12.1)	17 (30.9)	
Family	8 (12.1)	6 (10.9)	
Poverty	9 (13.6)	6 (10.9)	
Other reason	8 (12.1)	6 (10.9)	

*p<0.05 for difference between genders.

The median age was 31 years for men and 30 years for women. Educational attainment showed variation by gender, with the majority of women having no formal education (52.8%; 38/53) while the majority of men had attended primary school grades 1–6 (51.5%; 35/68). Nearly one-third of men (29.4%; 20/68) but only 13.2% (7/53) of women reported post-secondary education. A majority of men were single, divorced, widowed, or separated (61.9%) and 38.1% were married/cohabiting; this trend was the reverse for women (30.0% single and 70.0% married/cohabiting). In terms of safety of dwelling, similar proportions of men (36.4%; 24/66) and women (42.6%; 23/54) reported that they did not have a safe place to sleep in the last month. Main reasons for traveling included war or conflict (33.3% among men; 16.4% among women) and other violence (16.7% among men and 20.0% among women). Women also frequently cited work (30.9% among women) as a reason for traveling.

### Recent sexual behavior, condom use, STI symptoms and testing

Results of descriptive analyses for sexual behaviors and reported violence are shown in Table 2. A total of 26.1% (18/69) men and 36.4% (20/55) women reported sexual intercourse in the past month. Of those, among men, 50.0% (9/18) reported to have one sex partner and 44.4% (8/18) 3 sex partners; among women, 90.0% (18/20) reported one sex partner and 10.0% (2/20) 3 sex partners. Last sex partner was reported to be casual (a stranger, a friend or travel companion, a person of authority, a sex worker, or another person other than a regular sex partner or husband/wife) among 43.0% (21/49) of men and 27.8% (10/36) of women and formal (a regular sex partner or husband/wife) among 57.1% (28/49) of men and 72.2% (26/36) women; of those, 42.9% (9/21) of men and 80.0% (8/10) of women reported not using a condom with their last casual partner. A total of 32.7% (18/55) of men and 18.2% (8/44) of women reported never having an HIV test prior to this study.

**Table 2:** Sexual behaviors, access to condoms, STI symptoms, and HIV testing (self-reported by adults age ≥18 years).

	Men, *n* (%)	Women, *n* (%)	*p*-value (difference between genders)
Age at first marriage/cohabitation	*n* = 56	*n* = 47	0.07
<18 years	21 (37.5)	26 (55.3)	
≥18 years	35 (62.5)	21 (44.7)	
Gender of sex partners in lifetime	*n* = 60	*n* = 46	0.95^a^
Only men	2 (3.3)	43 (93.5)	
Only women	53 (88.3)	0 (0.0)	
Men and women	3 (5.0)	2 (4.4)	
Transmen	0 (0.0)	0 (0.0)	
Transwomen	0 (0.0)	0 (0.0)	
With no one	2 (3.3)	1 (2.2)	
**Recent Sexual Behavior, Condom Use & Access**			
Have you had sexual intercourse in the last 4 weeks?	*n* = 69	*n* = 55	0.22
Yes	18 (26.1)	20 (36.4)	
Number of sex partners in the last 4 weeks	*n* = 18	*n* = 20	0.02*
1	(50.0)	18 (90.0)	
2	1 (5.6)	0 (0.0)	
3	8 (44.4)	2 (10.0)	
Relationship with all partners in last 4 weeks (mark all that apply)	*n* = 14	*n* = 7	0.03*
Stranger	0 (0.0)	2 (10.5)	
Regular sex partner	1 (5.6)	0 (0.0)	
Spouse	10 (55.6)	4 (21.0)	
Friend or someone who is accompanying you on the trip	2 (11.1)	0 (0.0)	
Authority figure (e.g., a politician or a leader)	0 (0.0)	0 (0.0)	
Sex worker	1 (5.6)	0 (0.0)	
Other	0 (0.0)	1 (5.3)	
The last time you had sex (either anal or vaginal) did you use a condom?	*n* = 49	*n* = 36	
*Among those who reported last sex was with a casual partner:*	*n* = 21	*n* = 10	0.05
Did not use condom	9 (18.4)	8 (22.2)	
Yes, used condom	12 (24.5)	2 (5.6)	
*Among those who reported last sex was with a formal partner:*	*n* = 28	*n* = 26	0.76
Did not use condom	15 (30.6)	15 (41.7)	
Yes, used condom	13 (26.5)	11 (30.6)	
Do you feel like since you left your country of origin or last country of residence, it is harder to access condoms?	*n* = 53	*n* = 37	0.16
No	31 (58.5)	15 (40.5)	
Yes	10 (18.9)	7 (18.9)	
Does not apply	12 (22.6)	15 (40.5)	
STI symptoms in past month	*n* = 52	*n* = 40	<0.01*
Unusual discharge from the penis or vagina	0 (0.0)	6 (15.0)	
Sores or warts on the genital area	3 (5.8)	0 (0.0)	
Painful or frequent urination	1 (1.9)	3 (7.5)	
Itching and redness in the genital area	1 (1.9)	3 (7.5)	
Abnormal vaginal odor	0 (0.0)	3 (7.5)	
Anal itching, soreness, or bleeding	0 (0.0)	0 (0.0)	
Abdominal pain	0 (0.0)	6 (15.0)	
Enlarged lymph nodes in the groin area	0 (0.0)	0 (0.0)	
None of the above symptoms	48 (92.3)	22 (55.0)	
Date of last HIV test	*n* = 55	*n* = 44	0.19
Within the last year	16 (29.1)	21 (47.7)	
More than 1 year ago	15 (27.3)	13 (29.6)	
Never	18 (32.7)	8 (18.2)	
I don’t know	4 (7.3)	2 (4.6)	
Prefer not to say	2 (3.6)	0 (0.0)	
Other than HIV, have you ever received treatment for another STI (gonorrhoeae, chlamydia, syphilis, herpes, trichomonas)	*n* = 54	*n* = 42	0.58
Within the last year	4 (7.4)	4 (9.5)	
More than 1 year ago	7 (13.0)	1 (2.4)	
Never	39 (72.2)	32 (76.2)	
I don’t know	2 (3.7)	2 (4.8)	
Prefer not to say	2 (3.7)	3 (7.1)	

*p<0.05 for difference between genders.

^a^ comparison calculated with response categories opposite sex; same sex; men and women; no one.

Clinical screening (data not shown) revealed that of those screened, 58.8% (30/51) of women and 5.7% (4/70) of men reported a current reproductive tract infection symptom. Among women screened, vaginal discharge was the most common symptom (40.0%; 12/30). Of those reporting vaginal discharge, 25.0% tested positive for leukocyte esterase in urine; 36.4% were positive for Amsel criteria; and 45.4% had hyphae/pseudohyphae evocative of *C. albicans* upon KOH evaluation using microscopy. Laboratory testing revealed that of 78 men, HIV and syphilis were found among 1.3% (*n* = 1) and 2.6% (*n* = 2) respectively. Among 63 women tested, 3.2% (*n* = 2) were positive for HIV and 3.2% (*n* = 2) were positive for syphilis. All HIV positive samples were identified as advanced HIV disease, with CD4 counts <200 cells/mm^3^.

### Other STI vulnerabilities prior to and during migration: transactional sex and concurrent partnerships

Results of descriptive analyses for transactional sex and concurrent partnerships are shown in Table 3. About thirteen percent (12.7%; 7/55) of men and 8.3% (3/41) of women reported ever receiving money, goods, favors, presents, drugs, or housing in exchange for sex. More than 1 in 4 (26.8%; 15/56) men and 14.7% (5/34) of women reported having sex with someone who was not their formal partner while they were in a partnership; among these, 35.7% of men and 25.0% of women reported not using a condom with the secondary partner. Nearly all of these experiences occurred in the respondent's country of origin or country of residence (men: 92.9%, 13/14; women: 100%, 4/4). In addition, 7.0% (4/57) of men and 16.2% (6/37) of women reported having ever been in a relationship and their partner had another partner at the same time. Among men who reported a non-monogamous partner, this occurred in transit (66.7%; 2/3) or in a country of residence (33.3%; 1/3). Among women who reported a non-monogamous partner, this occurred before leaving country of origin (16.7%; 1/6), in transit (50.0%; 3/6), in country of residence (16.7%; 1/6), or in Panamá (16.7%; 1/6). There were no statistically significant differences in reports of these experiences by gender.

**Table 3:** Transactional Sex and concurrent partnerships (self-reported by adults age ≥18 years).

	Men, *n* (%)	Women, *n* (%)	*p*-value (difference between genders)
**History of Transactional Sex**			
When, if ever, have you received money, goods, favors, presents, drugs, or housing for sex?	*n* = 55	*n* = 41	0.20
Before leaving my last country of residence	4 (7.3)	1 (2.4)	
After leaving my last country of residence but before arriving in Panamá	3 (5.5)	0 (0.0)	
After I entered Panamá	0 (0.0)	1 (2.4)	
Both before leaving my country of residence and after leaving my country of residence	0 (0.0)	1 (2.4)	
Never	48 (87.3)	38 (92.7)	
When, if ever, have you given money, goods, favors, presents, drugs, or housing for sex?	*n* = 55	*n* = 39	0.96
Before leaving my last country of residence	3 (5.5)	2 (5.1)	
After leaving my last country of residence but before arriving in Panamá	0 (0.0)	0 (0.0)	
After I entered Panamá	0 (0.0)	0 (0.0)	
Both before leaving my country of residence and after leaving my country of residence	2 (3.6)	1 (2.6)	
Never	50 (90.9)	36 (92.3)	
**History of Concurrent Partnerships**			
In your lifetime, have you had sex with someone who is not your formal partner, while you were in a partnership?	*n* = 56	*n* = 34	0.18
No	41 (73.2)	29 (85.3)	
Yes	15 (26.8)	5 (14.7)	
When was this?	*n* = 14	*n* = 4	0.83
Before leaving country of origin	9 (64.3)	3 (75.0)	
In transit to another country or to Panamá	1 (7.1)	0 (0.0)	
In a country of residence	4 (28.6)	1 (25.0)	
After arriving in Panamá while in the Gap or in the MRS	0 (0.0)	0 (0.0)	
Did you use a condom?	*n* = 14	*n* = 4	0.68
No	5 (35.7)	1 (25.0)	
Yes (during all or part of the act)	9 (64.3)	3 (75.0)	
Have you been in a relationship and your partner had another partner while you were with them?	*n* = 57	*n* = 37	0.16
No	53 (93.0)	31 (83.8)	
Yes	4 (7.0)	6 (16.2)	
When was it that your partner had sex with another person while you were with them?	*n* = 3	*n* = 6	0.83
Before leaving country of origin	0 (0.0)	1 (14.3)	
In transit to another country or to Panamá	2 (66.7)	3 (42.9)	
In a country of residence	1 (33.3)	1 (14.3)	
After arriving in Panamá while in the Gap or in the MRS	0 (0.0)	1 (14.3)	

### Sexual assault and perceived safety

Results about sexual assault and perceived safety are presented in Table 4. Of the 52 men and 45 women who responded about perceived safety from sexual assault, 40.4% (*n* = 21) of men and 44.4% (*n* = 20) of women felt somewhat or completely unsafe *before* leaving their last country of residence. Of men, 42.3% (*n* = 22) and 51.2% (*n* = 22) of women felt unsafe *while traveling from their last country of residence* before arriving in Panamá and 40.4% (*n* = 21) men and 54.8% (*n* = 23) of women felt unsafe from sexual assault *while traveling through the DF*.

**Table 4:** Perceived safety from sexual assault and history of sexual assault (self-reported by adults age ≥18 years).

	Men, *n* (%)	Women, *n* (%)	*p*-value (difference between genders)
Before leaving country of residence, how safe did you feel from sexual assault in your everyday life?	*n* = 52	*n* = 45	0.58
Not at all safe	14 (26.9)	13 (28.9)	
Somewhat unsafe	7 (13.5)	7 (15.6)	
Neither safe nor unsafe	5 (9.6)	1 (2.2)	
Completely safe	22 (42.3)	22 (48.9)	
It varies by the situation	4 (7.6)	2 (4.4)	
Since you left your last country of residence, how safe have you felt from sexual assault in your everyday life?			0.50
Not at all safe	14 (26.9)	17 (39.5)	
Somewhat unsafe	8 (15.4)	5 (11.6)	
Neither safe nor unsafe	4 (7.7)	5 (11.6)	
Completely safe	20 (38.5)	14 (32.6)	
It varies by the situation	6 (11.5)	2 (4.6)	
Since you arrived in Panamá, how safe have you felt from sexual assault in your everyday life?	*n* = 52	*n* = 42	0.62
Not at all safe	14 (26.9)	15 (35.7)	
Somewhat unsafe	7 (13.5)	8 (19.0)	
Neither safe nor unsafe	4 (7.7)	3 (7.1)	
Completely safe	21 (40.4)	14 (33.3)	
It varies by the situation	6 (11.5)	2 (4.8)	
Lifetime sexual assault or rape	*n* = 54	*n* = 45	0.49
Yes, more than one time	1 (1.8)	4 (8.9)	
Yes, once	4 (7.4)	2 (4.4)	
No, this has not happened to me	45 (83.3)	34 (75.6)	
Dońt know/cannot remember	2 (3.7)	3 (6.7)	
I prefer not to say	2 (3.7)	2 (4.4)	
Age of first rape or sexual assault (years)	*n* = 5	*n* = 6	
	median: 20 (IQR 16–24)	median: 17 (IQR 15–19)	0.28
When did this happen?			0.50
Before leaving my country of origin	3 (60.0)	4 (66.7)	
In another country of residence	1 (20.0)	2 (33.3)	
In transit to Panamá	0 (0.0)	0 (0.0)	
While in Panamá (on the trail or at an ERM)	0 (0.0)	0 (0.0)	
I prefer not to say	1 (20.0)	0 (0.0)	

Participants were also asked about sexual assault (being “forced or frightened by another person into doing something sexually that you did not want to do”). Of those who reported lifetime sexual assault, 60.0% (*n* = 3) of men and 66.7% (*n* = 4) of women reported that this occurred in their last country of residence; 0.0% of respondents reported sexual assault during migration or during their time in Panamá.

### Pregnancy history and contraceptive use

Finally, results of pregnancy testing and questions about pregnancy are shown in Table 5. A total of 20.0% (*n* = 11) of women tested positive for pregnancy; two women did not know of their pregnancy prior to testing. Five of the 11 pregnancies were planned. Of pregnant women, 72.7% (*n* = 8) had one sex partner in the past year, and 27.3% (*n* = 3) had 2 or more partners; the last sexual encounter of pregnant women was within the last 28 days for 36.4% (*n* = 4) and 54.5% (*n* = 6) > 28 days. Among women, 39.2% (20/51) were using contraceptives at time of study.

**Table 5:** Pregnancy (self-reported by adults age ≥18 years).

	Men, *n* (%)	Women, *n* (%)	*p*-value (difference between genders)
Lifetime number of pregnancies (men: number of times you impregnated a woman)	*n* = 46	*n* = 46	0.14
0	13 (28.3)	7 (15.2)	
1	16 (34.8)	10 (21.7)	
2	8 (17.4)	11 (23.9)	
3	5 (10.9)	8 (17.4)	
4 or more	4 (8.7)	10 (21.7%)	
Age at first pregnancy (women)	*n* = 31	*n* = 34	0.42
<18 years	4 (12.9)	8 (23.5)	
18–30 years	22 (71.0)	23 (67.6)	
>30 years	5 (16.3)	3 (8.8)	
Are you currently pregnant?		*n* = 39	
No		30 (76.9)	
Yes^a^		9 (23.1)	
How many months pregnant are you?		*n* = 8	
First trimester		4 (50.0)	
Second trimester		2 (25.0)	
Third trimester		2 (25.0)	

^a^ 9 women self-reported that they were currently pregnant; blood testing confirmed pregnancy among 11 women.

## Discussion

Our study showed high recent sexual activity, low condom use with casual partners, and a need for increased access HIV and syphilis testing and treatment. In addition, we found high prevalence of reproductive tract symptoms, particularly among women. Taken together, these findings indicate a need for increased testing, condom provision, and SRH healthcare access at Migrant Reception Stations that receive migrant peoples in transit in Panamá.

The findings of this study should be considered in light of several limitations. First, this was a rapid epidemiological assessment conducted over a short period of time in January 2022. There is often a lull in migration flow through Panamá around the December and January holidays. However, COVID-19 slowed migration in 2020, followed by a boom of people passing throughout most of 2021, and a lull again in December 2021. Migrant peoples also anticipated policy changes affecting migrants and immigrants to Mexico and the US in early 2022. As a result, the MRS received between 35 and 250 migrants per day at the time of data collection in January 2022. In addition, this study focused on migrant peoples in transit; there may be different SRH needs at different stages of migration: predeparture, in transit, interception, and arrival at destination (20). Finally, our self-applied questionnaire allowed participants to skip questions that they did not wish to answer, and some items had large proportions of missing data (e.g., only 7/20 women who reported sexual intercourse in the last 4 weeks answered the item on the relationship to that sexual partner). Missing data could bias results if respondents with particular characteristics were less willing to respond.

The UN Committee on Economic, Social, and Cultural Rights and the Convention on the Elimination of All Forms of Discriminations against Women affirm that sexual and reproductive health and access to SRH services are basic human rights (21). Indeed, the 2030 Sustainable Development Goals include the aim to achieve access to universal SRH services (22). However, access to SRH services remains limited in many low and middle income countries and in humanitarian settings (3), often due to scarce resources and inadequate infrastructure (7, 23).

A recent systematic review demonstrated that there are a number of evidence-based interventions to improve HIV and STI prevention knowledge and contraception and condom-use skills for young people in LMIC and humanitarian settings, and that these require adaptation “to the context and realities of humanitarian settings (such as high levels of trauma exposure and loss),” (3). The authors suggest that in such resource-limited and challenging settings, trauma-informed approaches may be considered a priority rather than resource-demanding high intensity interventions. They also suggest that program planners should consider whether SRH services in LMIC and humanitarian settings could be integrated into existing service systems (3).

The Inter-Agency Field Manual on Reproductive Health in Humanitarian Settings provides an outline of minimum reproductive health interventions that should be implemented when a humanitarian crisis emerges (4). Of critical importance is the need for gender-specific care, as women and girls in humanitarian crises have distinct needs when engaging with SRH services (24). In the case of migration through the DF in Panamá, the humanitarian crisis is not new, nor is an end in sight. To address the SRH needs of highly vulnerable migrant peoples who transit the DF en route to North America, a minimum standard of integrated sexual and reproductive health interventions is necessary. Furthermore, the design and delivery of SRH services must take a regional approach, with participation from countries across Latin America and the Caribbean.

## Data Availability

The raw data supporting the conclusions of this article will be made available by the authors, without undue reservation.
